# Oil Recovery by Synergistic CO_2_ Flooding Using Viscoelastic Fluids of Low Interfacial Tension Containing Amphiphilic Nanoparticles

**DOI:** 10.3390/molecules31193493

**Published:** 2026-10-01

**Authors:** Mengsheng Jiang, Jilong Zheng, Chuan He, Shanfa Tang, Yuanwu Dong, Zhe Wang

**Affiliations:** 1 School of Petroleum Engineering, Yangtze University, Wuhan 430100, China; 2 National Key Laboratory of Low-Carbon Catalysis and CO_2_ Utilization, Yangtze University, Wuhan 430100, China; 3 Hubei Key Laboratory of Oil and Gas Drilling and Production Engineering, Yangtze University, Wuhan 430100, China; 4 Research Institute of Oil and Gas Technology, Tarim Oilfield Company, Korla 841000, China

**Keywords:** amphiphilic nanoparticles, low-interfacial-tension viscoelastic fluid, CO_2_ flooding, gas channeling control

## Abstract

Tight-oil reservoirs are abundant but characterized by poor physical properties and strong heterogeneity. CO_2_ flooding is an important technique for enhanced oil recovery (EOR) and carbon storage, yet CO_2_ channeling remains a critical issue due to its high mobility, and existing anti-channeling methods have limitations. In this work, a low-interfacial-tension viscoelastic fluid containing amphiphilic nanoparticles (LFNA) was formulated by combining a small-molecule viscosifier (LFLM) with amphiphilic nanoparticles (LQNA). Its performance and synergistic effect with CO_2_ flooding were systematically evaluated. The LFNA formulation is simple (0.4 wt% LFLM + 0.01 wt% LQNA-3), exhibiting a viscosity of 5.82 mPa·s, an interfacial tension of 0.0296 mN/m, and predominantly elastic behavior (tan δ = 0.791). It shows good tolerance to temperature, salinity, and pH variation, favorable injectivity in tight cores (Kg = 0.45 − 1.07 mD), and selective plugging capability. Core-flooding experiments in both homogeneous and heterogeneous models demonstrate that CO_2_–LFNA alternating injection after waterflooding outperforms continuous CO_2_ flooding in EOR. In heterogeneous models, the LFNA slug preferentially plugs high-permeability channels, expands sweep volume, and effectively mobilizes residual oil in low-permeability zones, with incremental recoveries of 12.49% and 15.74% for low- and high-permeability cores, respectively. Overall, the combined incremental recovery of CO_2_–LFNA alternating flooding (14.26%) exceeds that of water–LFNA alternating flooding (11.49%) and continuous CO_2_ flooding (9.85%). These results confirm that LFNA provides a synergistic effect of plugging, oil-washing, and sweep improvement. Using LFNA for CO_2_ channeling control is feasible and effective, and it also benefits CO_2_ storage and carbon reduction.

## 1. Introduction

With the advancement of oil and gas exploration and development in China, tight-oil reservoirs have become a primary development target due to their abundant reserves. However, their poor physical properties (Kg ≤ 1 mD, pore size ≤ 1 μm), strong heterogeneity, and insufficient formation energy result in low production under natural depletion, requiring reservoir energizing to enhance oil mobility and recovery [1,2]. CO_2_ flooding is an important technical approach for enhancing oil recovery in tight reservoirs while achieving effective CO_2_ storage and carbon reduction [3]. Nevertheless, due to poor reservoir properties, strong interlayer heterogeneity, and CO_2_’s high mobility, severe gas channeling often occurs, compromising both oil recovery and storage effectiveness [4]. Existing anti-channeling technologies, such as WAG flooding, foam flooding, polymer gels, and small-molecule amine reactions with CO_2_, all have shortcomings [5,6,7,8]. Therefore, there is an urgent need to develop and evaluate a relatively simple anti-channeling system suitable for heterogeneous tight reservoirs, with good injectivity, oil-washing capability, and adaptability to reservoir conditions.

Low-interfacial-tension viscosifying small-molecule fluids exhibit functions similar to surfactant/polymer binary flooding. Due to their low molecular weight, they can meet the injectivity and sweep efficiency requirements of heterogeneous tight reservoirs while improving oil-washing efficiency, achieving incremental recoveries of 10–20% [9]. However, their temperature resistance and viscosifying performance require improvement. In recent years, amphiphilic nanoparticles have demonstrated superior interfacial properties, dispersion stability, smaller particle sizes (1.0–100 nm), lower dosages (0.005–0.01%), and temperature/salt resistance compared to conventional nanoparticles [10,11]. They can be dispersed in solvents of varying polarity, exhibiting favorable interactions with gas (CO_2_) and liquids (oil/water), and can enhance viscosity [12]. Accordingly, to overcome the gas channeling challenges in CO_2_ flooding of heterogeneous tight reservoirs, this work utilizes low-interfacial-tension viscosifying small molecules and amphiphilic nanomaterials to construct an amphiphilic nanoparticle-containing low-interfacial-tension viscoelastic fluid. The environmental adaptability, injectivity, and CO_2_ flooding performance improvement under heterogeneous conditions were systematically evaluated for the target tight reservoir (strongly heterogeneous, high salinity, relatively high temperature). This provides a new approach for CO_2_ flooding anti-channeling technology research.

## 2. Literature Review

Several technical approaches have been reported to mitigate CO_2_ gas channeling in heterogeneous tight-oil reservoirs, each with distinct practical constraints. Foam systems rely on gas–liquid lamellae to increase apparent gas-phase viscosity, yet they are vulnerable to high salinity, reservoir temperature, and crude-oil contamination, leading to rapid lamella rupture and performance loss [6]. Cross-linked polymer-based gel agents can form high-strength blocking barriers for thief zones; however, high-molecular-weight gel precursors suffer poor injectivity in tight nanosized pore throats, and divalent ions in formation water often interfere with gelation behavior [4]. CO_2_-responsive worm-like micelle systems realize in situ viscosity build-up upon contacting CO_2_, but their performance window is narrow and highly sensitive to fluctuations in reservoir brine composition [13]. Conventional water-alternating-gas (WAG) improves mobility ratio to a limited extent, but cannot fundamentally solve preferential CO_2_ breakthrough in strongly heterogeneous tight formations [3].

Nanoparticle-assisted chemical agents represent an emerging branch for CO_2_ mobility-control research, with varied material designs reported in recent years. Inorganic nanoparticles can reinforce surfactant or micelle assemblies, but tend to aggregate under high-salinity reservoir conditions without surface modification [14,15]. Amphiphilic nanoparticles with dual hydrophilic–hydrophobic moieties exhibit better brine compatibility and interfacial activity; they can act as physical cross-linkers to strengthen small-molecule micellar networks, improving rheological stability without sacrificing injectivity [11,12]. Nevertheless, most existing amphiphilic-nanoparticle composite formulations are mainly evaluated for waterflooding scenarios. Their integrated performance, including anti-channeling capacity, oil-washing efficiency, and multi-condition tolerance, has rarely been systematically examined under real CO_2_-alternating displacement conditions for tight-oil reservoirs [16]. This creates the research niche for the LFNA investigated in the present study.

## 3. Results

### 3.1. Construction and Performance of Amphiphilic Nanoparticle-Containing Low-Interfacial-Tension Viscous Fluid

**Figure 1:**
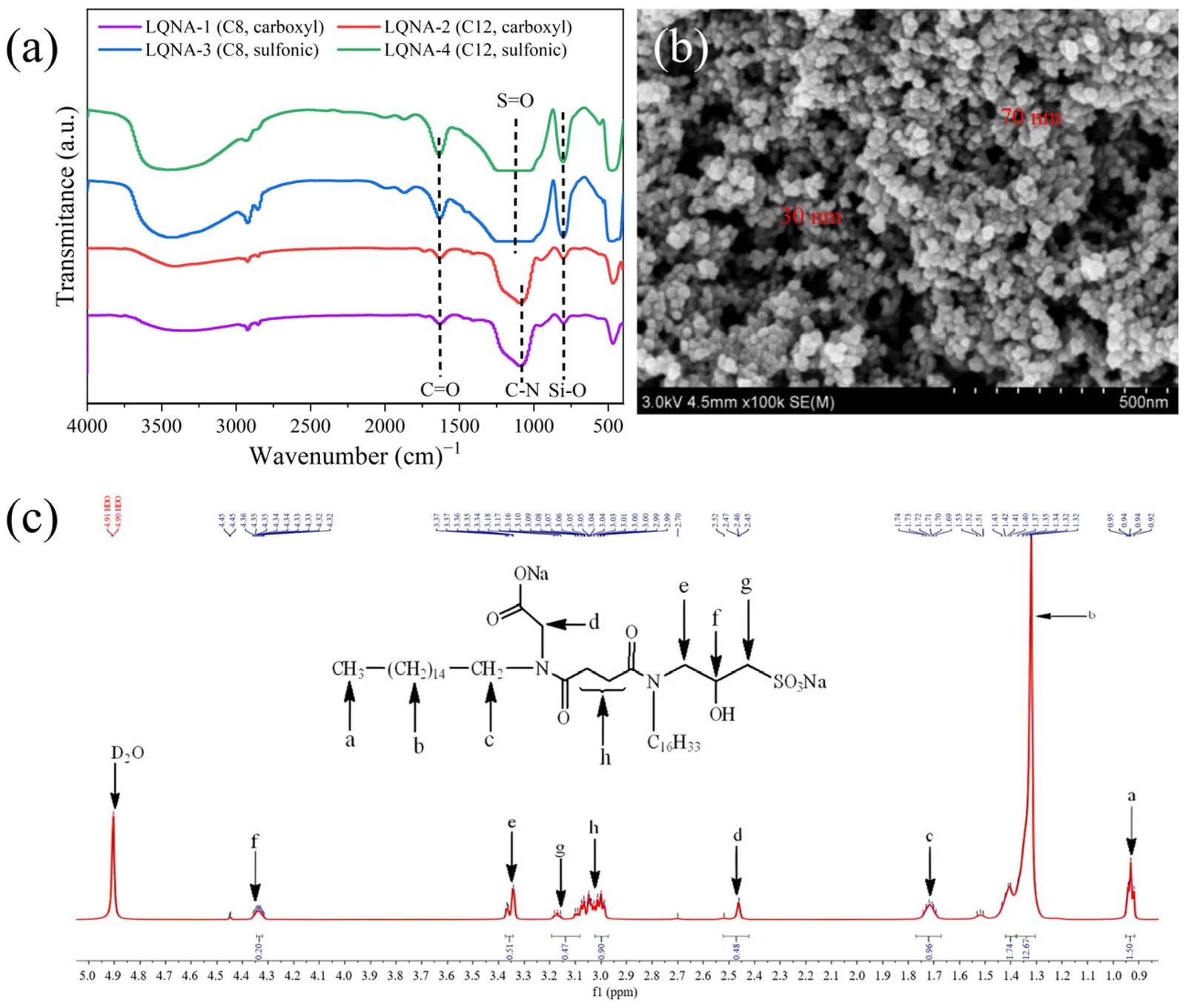
Material characterizations of amphiphilic nanoparticles and small-molecule viscosifier LFLM. (**a**) FT-IR spectra of LQNA-1 (C8, carboxyl), LQNA-2 (C12, carboxyl), LQNA-3 (C8, sulfonic), and LQNA-4 (C12, sulfonic); (**b**) SEM micrograph of surface-modified nano-silica; (**c**) ^1^H-NMR spectrum of small-molecule viscosifier LFLM measured in D_2_O.

Fourier-transform infrared (FT-IR) spectroscopy was performed on four modified nano-silica samples, namely LQNA-1 (C8, carboxyl), LQNA-2 (C12, carboxyl), LQNA-3 (C8, sulfonic), and LQNA-4 (C12, sulfonic) (Figure 1a). All samples exhibited the stretching vibration of Si-OH groups in the range of 3337–3460 cm^−1^. Characteristic absorption bands assigned to methyl and methylene groups originating from silane coupling agents were observed at 2852–2924 cm^−1^, while the symmetric stretching vibration of Si-O-Si bonds appeared around 800 cm^−1^, demonstrating that the silica matrix retained its structural integrity and hydrophobic alkyl chains were successfully incorporated onto the surface. Characteristic stretching bands of C=O and C-N bonds detected in carboxyl-functionalized LQNA-1 (C8, carboxyl) and LQNA-2 (C12, carboxyl) verified effective grafting of carboxyl groups, and typical absorption signals corresponding to S=O, S-O, and C-N vibrations for sulfonic-group-modified LQNA-3 (C8, sulfonic) and LQNA-4 (C12, sulfonic) confirmed the successful immobilization of sulfonic groups on nano-silica. The SEM micrograph in Figure 1b shows the microscopic morphology of modified nano-SiO_2_ particles. Figure 1c displays the ^1^H NMR spectrum of LFLM, which acts as a small-molecule viscosifier to construct the viscoelastic network of the composite fluid, and its proton signals match well with the labeled molecular structure.

The effects of a series of amphiphilic nanoparticles on LFLM solution viscosity and interfacial tension are shown in Table 1. The introduction of amphiphilic nanoparticles favors viscosity enhancement of the low-interfacial-tension small-molecule viscosifying system. With the same hydrophilic groups, increasing the hydrophobic carbon-chain length of the amphiphilic nanoparticles slightly reduces viscosity. With the same hydrophobic chain length, sulfonic acid-based nanoparticles exhibit better viscosifying performance than carboxylic acid-based ones; LQNA-3 shows the best viscosifying effect. The hydrophilic/hydrophobic balance of the nanoparticles has a minor effect on the interfacial activity of LFLM solution. Due to the abundant hydrophilic groups (hydroxyl, carboxyl, sulfonic) and long hydrophobic chains on the nanoparticle surface, they not only possess surface-active characteristics but also form electrostatic and hydrogen-bonding interactions with small-molecule viscosifiers, creating a structural network that mitigates the negative effects of high temperature on viscosity, macroscopically manifested as increased fluid viscosity [17,18].

The effect of LQNA-3 concentration on LFLM solution viscosity, interfacial activity, and viscoelasticity was further investigated (Table 2). The optimal viscosifying effect is achieved at 0.01% LQNA-3, with minimal impact on interfacial activity, maintaining low interfacial tension (≤3.025 × 10^−2^ mN/m). LQNA-3 concentration affects viscoelasticity, with slight enhancement of elasticity (tan δ < 1). Thus, 0.01 wt% LQNA-3 combined with 0.4 wt% LFLM was selected to formulate the amphiphilic nanoparticle-containing low-interfacial-tension viscoelastic fluid, designated as LFNA. Hydrophilic groups of sulfonic acids deliver stronger electrostatic interactions compared with carboxyl groups; increasing hydrophobic carbon-chain length weakens intermolecular association, thus slightly reducing the viscosity of composite fluids. Minor variation in these surface groups hardly disturbs the interfacial activity of the assembled system.

### 3.2. Reservoir Adaptability of Amphiphilic Nanoparticle-Containing Low-Interfacial-Tension Viscoelastic Fluid (LFNA)

#### 3.2.1. Salinity Adaptability

**Figure 2:**
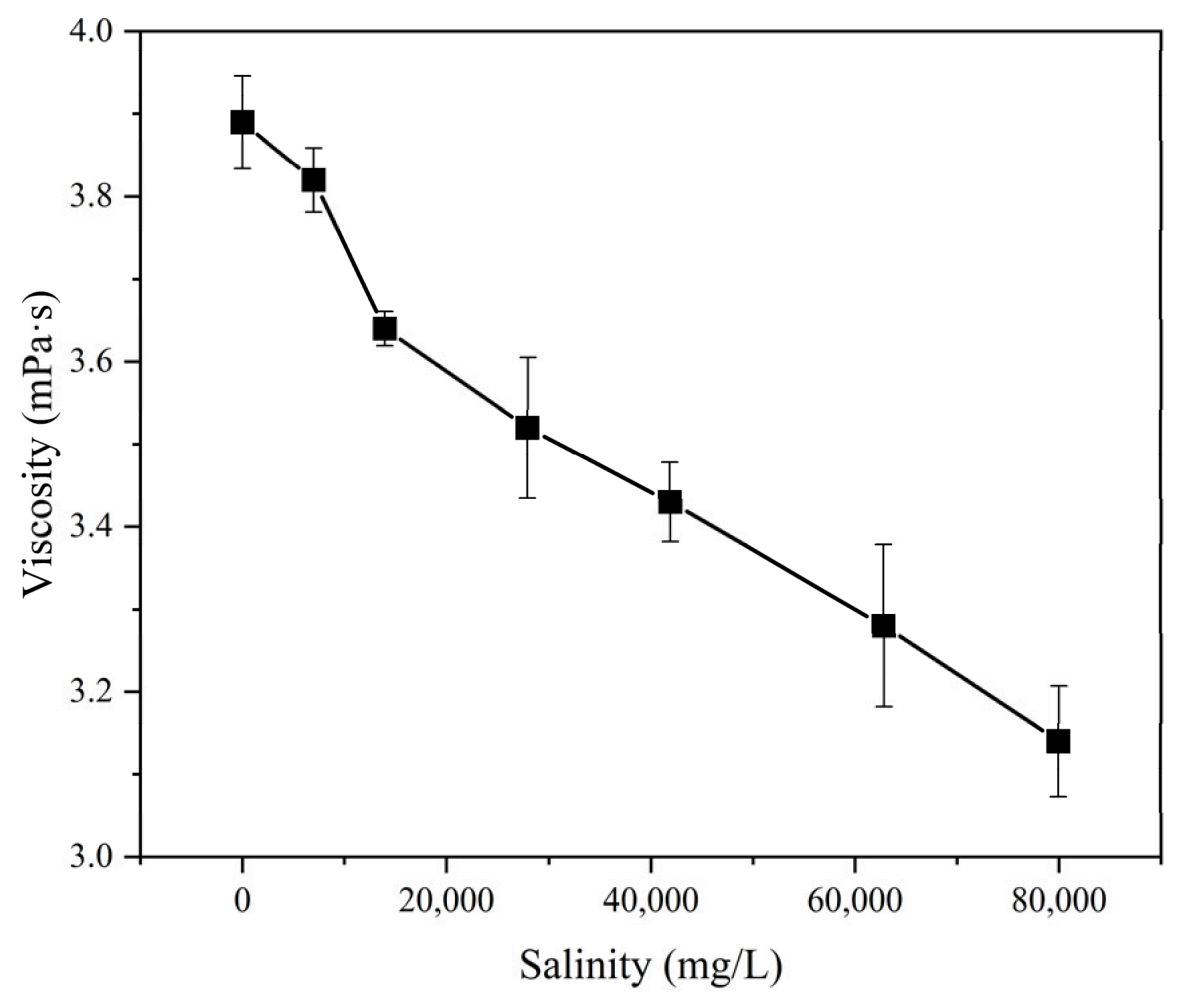
Effect of salinity on the viscosity of LFNA (at 47 °C).

**Figure 3:**
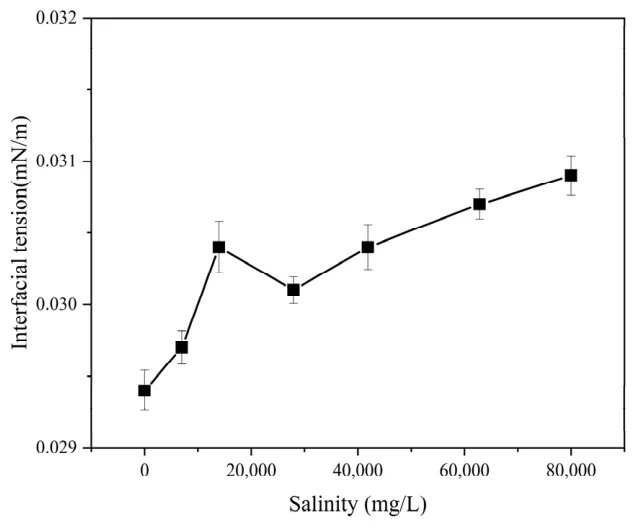
Effect of salinity on the interfacial tension of LFNA.

The effects of salinity (0–80,000 mg/L) on LFNA viscosity, oil/water IFT, and viscoelasticity are shown in Figure 2 and Figure 3 and Table 3. The data indicate that salinity (≤80,000 mg/L) has a minor effect on LFNA viscosity, interfacial activity, and viscoelasticity. Viscosity ranges from 4.59 to 5.93 mPa·s, IFT remains in the low range (2.94–3.09 × 10^−2^ mN/m), and tan δ ranges from 0.82 to 0.95, maintaining viscoelasticity with predominantly elastic behavior. In summary, salinity does not significantly affect LFNA performance, demonstrating good adaptability up to 80,000 mg/L [19]. Viscosity decreases slightly with increasing salinity while interfacial tension rises moderately, which is attributed to weak screening effects of inorganic ions on intermolecular non-covalent interactions within the LFNA network.

#### 3.2.2. pH Adaptability

**Figure 4:**
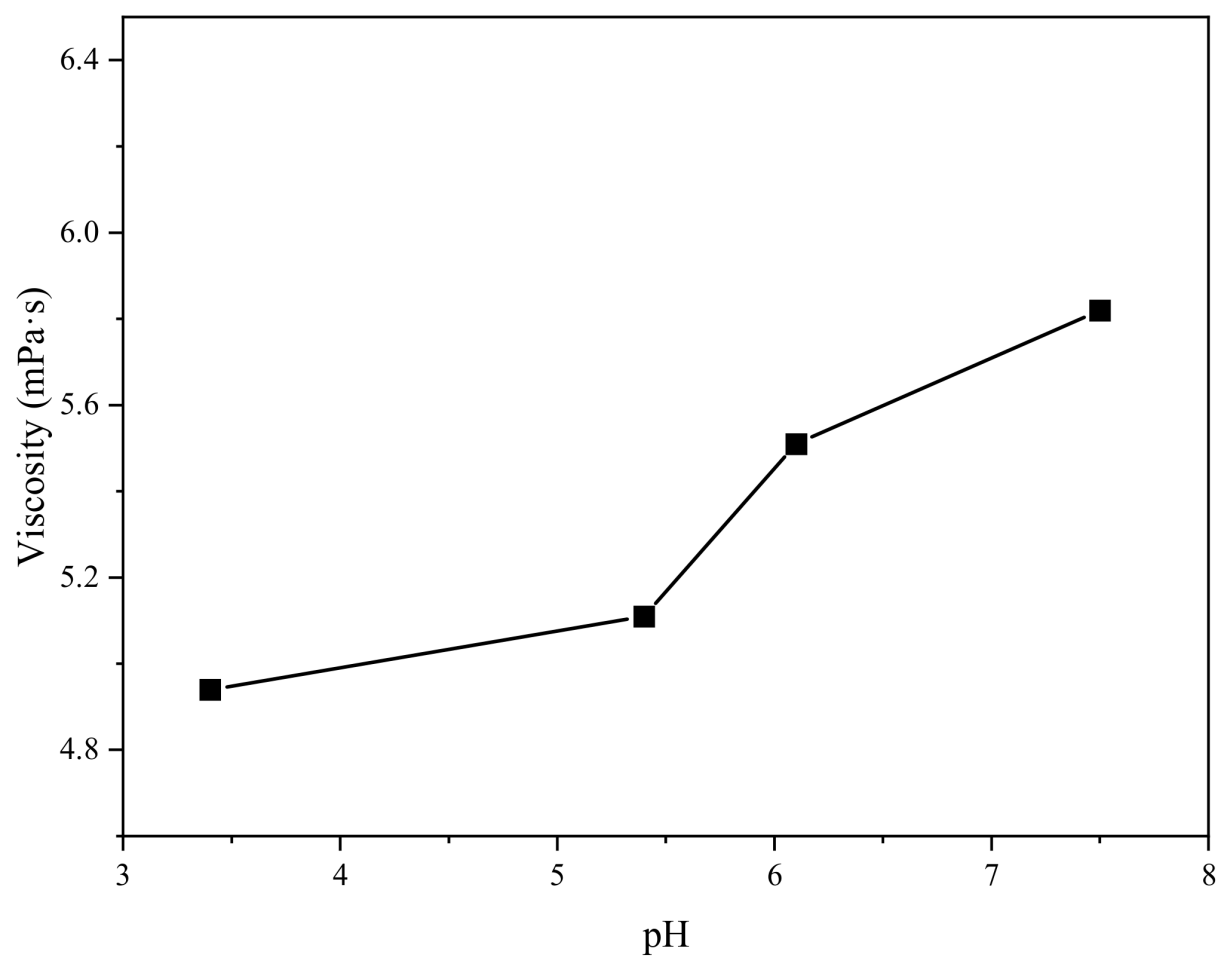
Effect of pH on the viscosity of LFNA.

**Figure 5:**
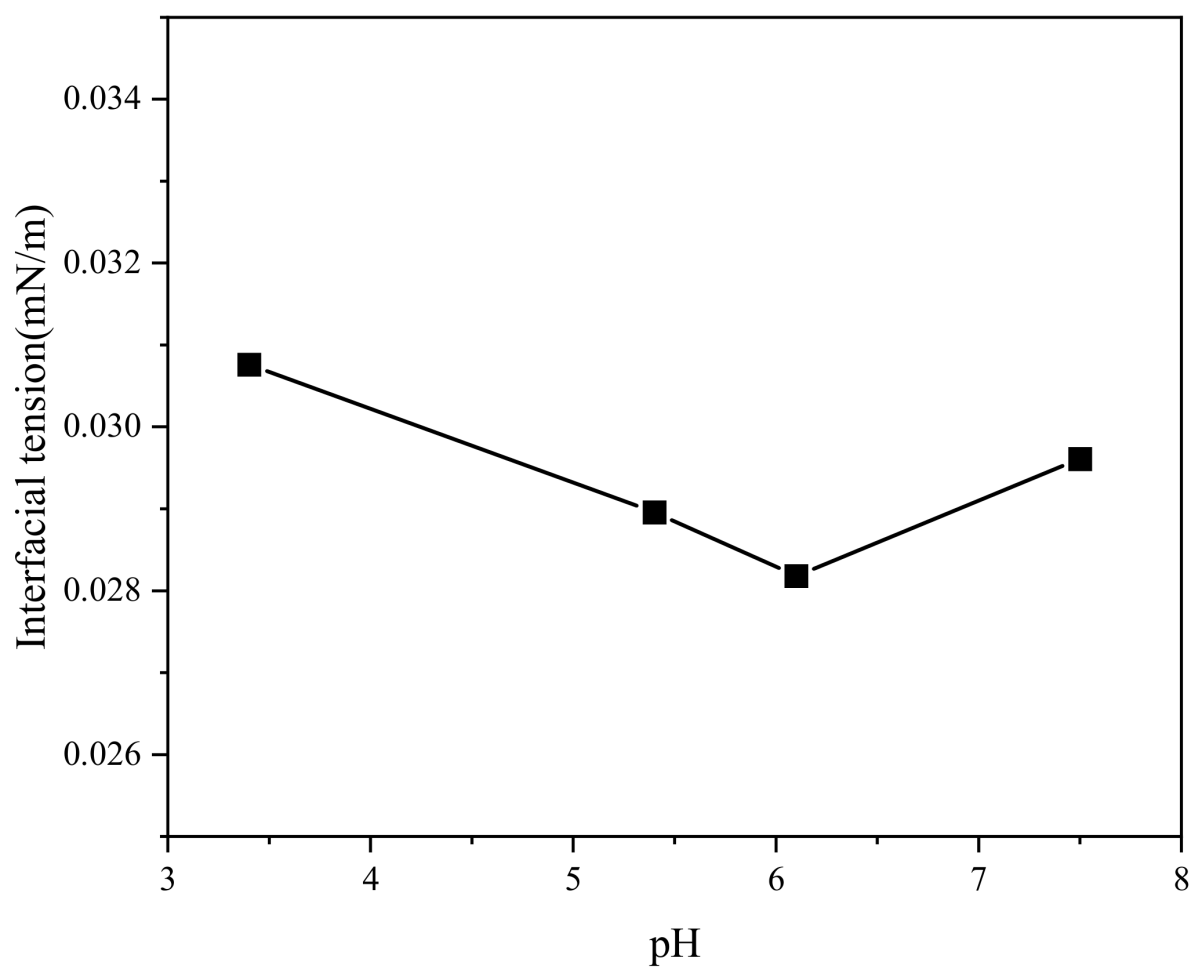
Effect of pH on the interfacial tension of LFNA.

LFNA viscosity, oil/water IFT, and viscoelasticity were measured at pH values of 3.4–7.5 (Figure 4 and Figure 5 and Table 4).

The results show that pH (3.4–7.5) has little effect on viscosity, IFT, or viscoelasticity. As pH decreases, viscosity and elasticity decline slightly, while IFT first decreases then increases, remaining in the low range (2.82–3.08 × 10^−2^ mN/m). Thus, the pH drop caused by CO_2_ dissolution in saline water has minimal impact on LFNA performance [20].

#### 3.2.3. Temperature Adaptability

**Figure 6:**
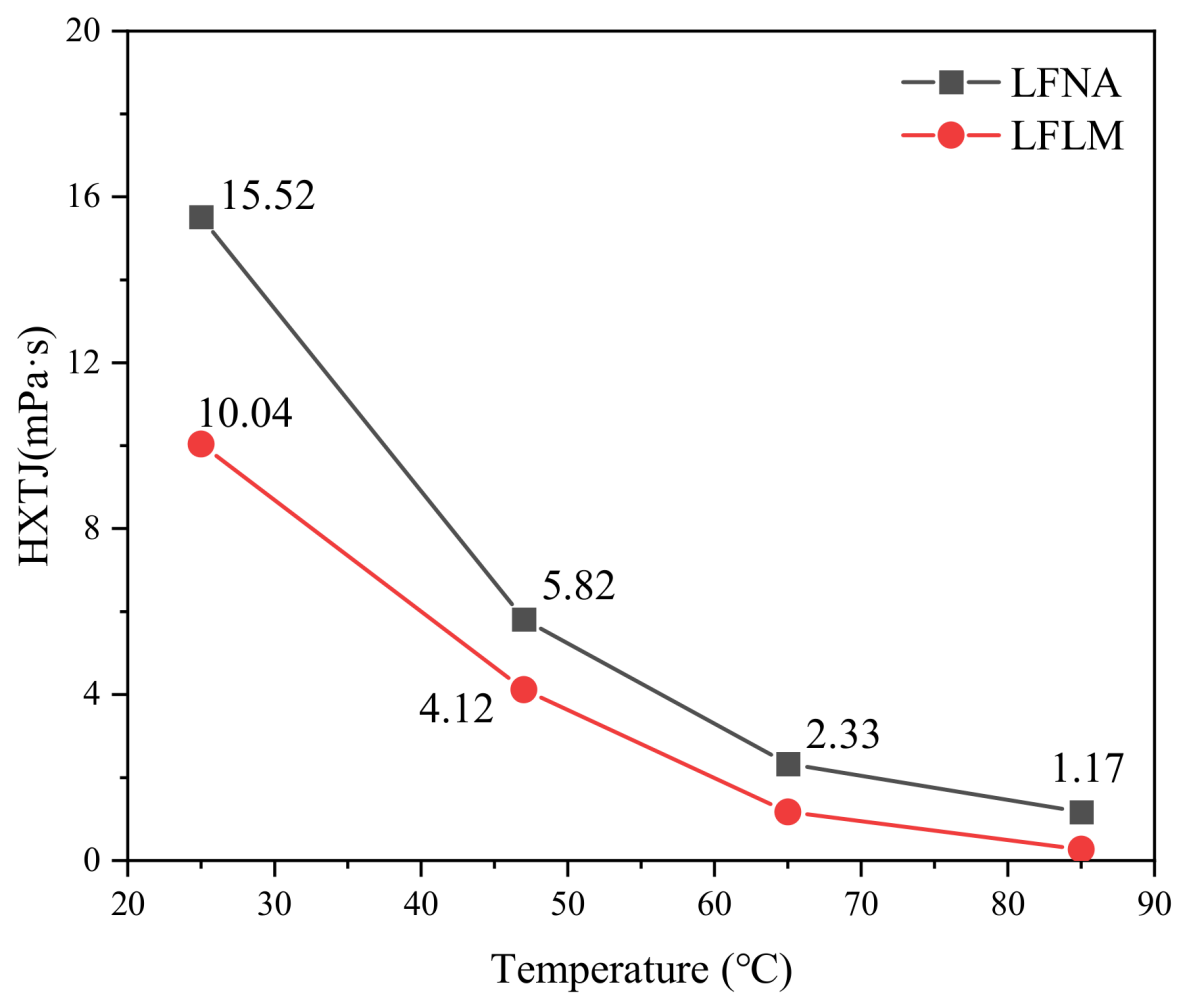
Effect of temperature on the viscosity of LFNA.

**Figure 7:**
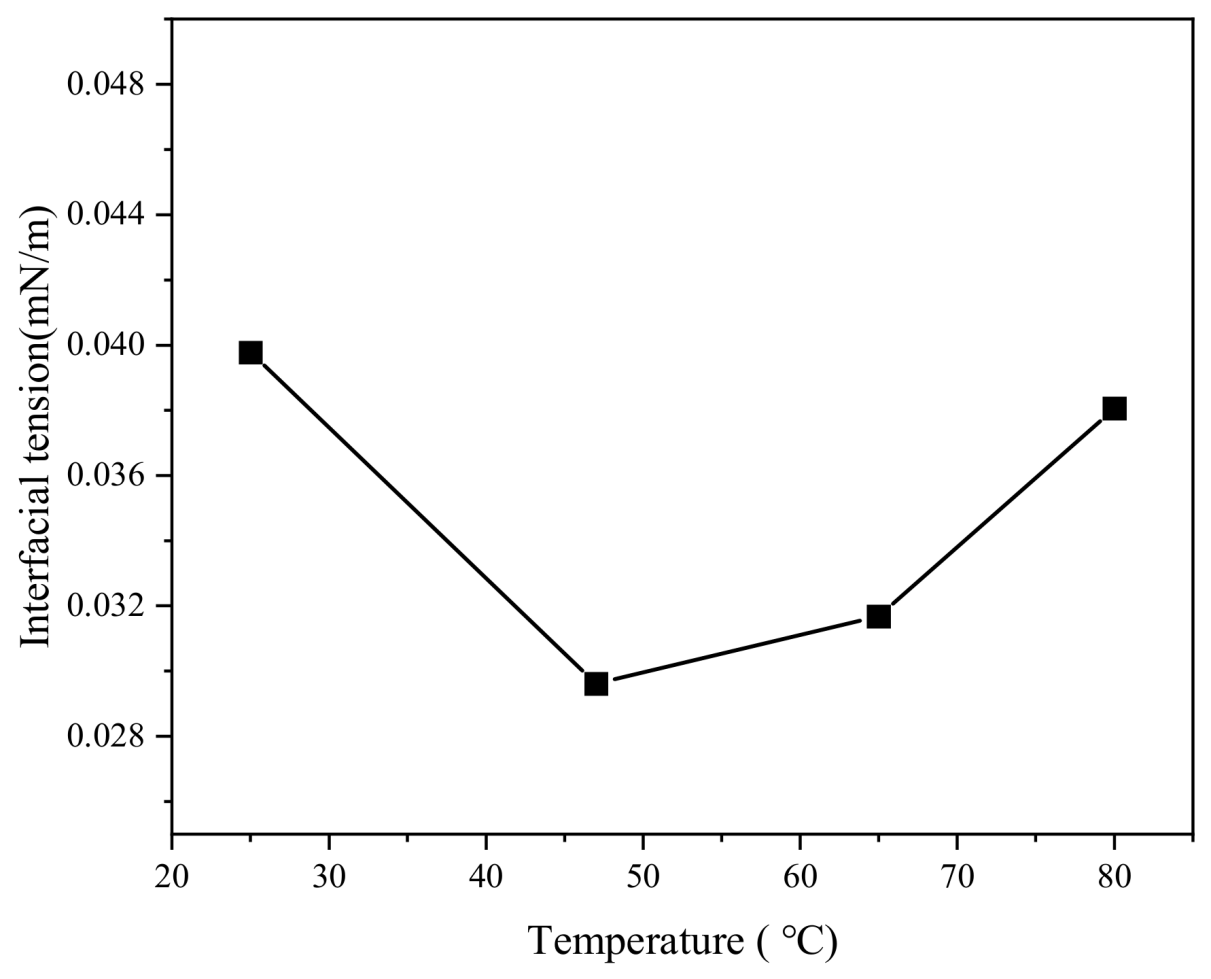
Effect of temperature on the interfacial tension of LFNA.

LFNA viscosity, oil/water IFT, and viscoelasticity were measured at 25–85 °C (Figure 6 and Figure 7 and Table 5).

With increasing temperature, LFNA viscosity decreases initially then levels off, but remains higher than LFLM solution alone. This is attributed to enhanced molecular thermal motion and reduced intermolecular interactions, while the presence of amphiphilic nanoparticles enhances network structure formation, mitigating temperature-induced viscosity loss [16]. IFT decreases initially then increases with temperature, but remains in the low range (2.96–3.98 × 10^−2^ mN/m). Viscoelasticity decreases with temperature, but tan δ remains <1 up to 85 °C, indicating predominantly elastic behavior.

In summary, within the experimental temperature range (25–80 °C), the interfacial activity and viscoelasticity of LFNA are not significantly affected, and it remains a low-interfacial-tension viscoelastic fluid. However, at higher temperatures, it is recommended to appropriately increase the concentration of LFNA to meet the viscosity requirements for field application.

#### 3.2.4. Core Injectivity Evaluation

**Figure 8:**
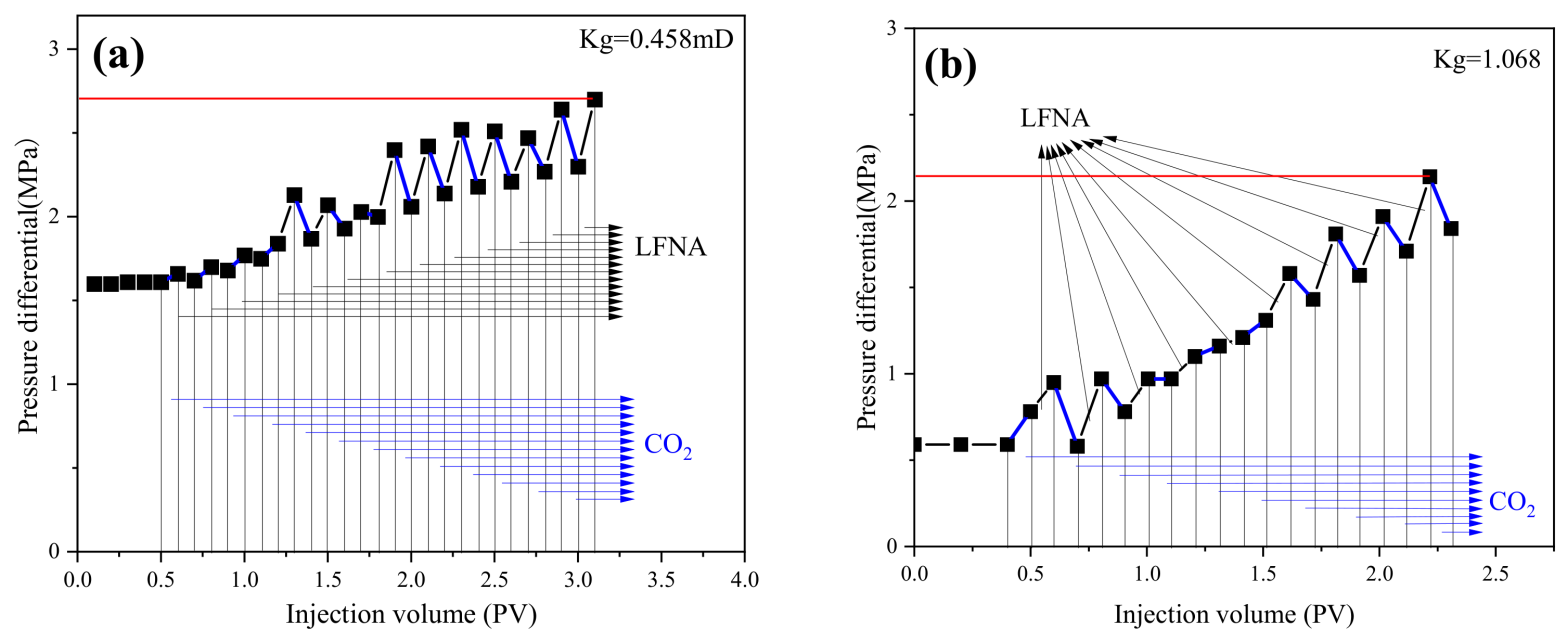
Injectivity results of CO_2_–LFNA alternating flooding in different tight cores. (**a**) Core permeability Kg = 0.45 mD; (**b**) core permeability Kg = 1.07 mD.

The injectivity of CO_2_–LFNA alternating injection in tight cores of varying permeability (0.45–1.07 mD) was evaluated at 65 °C (Figure 8).

After stable waterflooding, CO_2_–LFNA alternating flooding (0.1 PV slugs) was conducted. As alternation cycles and cumulative injection increased, injection pressure showed a stepwise increasing trend (with lower pressure during CO_2_ injection), without sudden pressure surges. At 1.8 PV cumulative injection, the maximum injection pressure differentials for both permeabilities (0.45 and 1.07 mD) were comparable and below 2.7 MPa, with slightly higher differentials for lower-permeability cores (by ~0.5 MPa). These results indicate good injectivity of CO_2_–LFNA alternating flooding across different permeabilities, with selective plugging capability [21]. The wavelike pressure variation reflects the dynamic balance between temporary flow resistance built by LFNA slug and flow re-opening upon subsequent CO_2_ injection.

### 3.3. Physical Model Flooding Performance Evaluation

#### 3.3.1. Homogeneous Model Flooding Experiments

**Figure 9:**
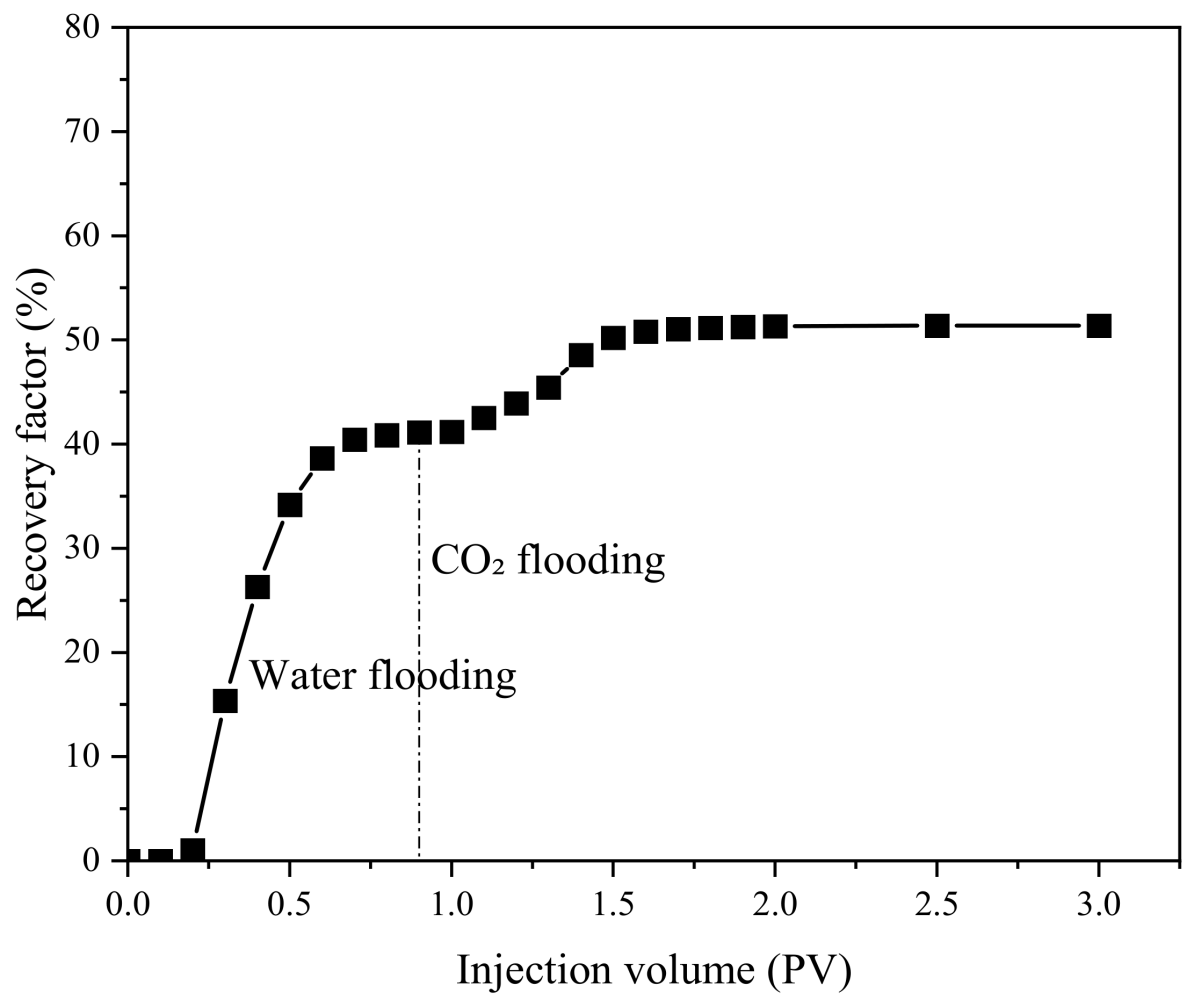
Enhanced oil recovery performance of continuous CO_2_ flooding after waterflooding.

**Figure 10:**
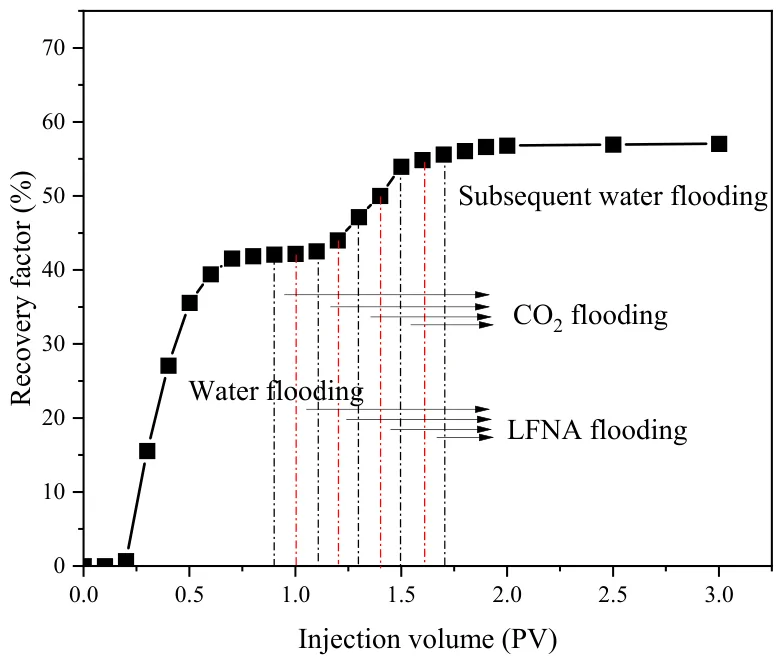
Oil recovery performance of CO_2_–LFNA alternating flooding (0.1 PV, 4 cycles) after waterflooding.

At 65 °C, continuous CO_2_ flooding and CO_2_–LFNA alternating flooding results are shown in Figure 9 and Figure 10, respectively.

Waterflood recoveries were 41.13% and 42.11%, respectively. After switching to continuous CO_2_ flooding (2.1 PV), final recovery reached 51.37%, an incremental 10.24%. In contrast, after waterflooding, CO_2_–LFNA alternating flooding (0.1 PV slugs, four cycles) achieved a total recovery of 55.63% at the end of the fourth cycle, an incremental 13.52% over waterflooding, with cycle increments of 0.41%, 4.64%, 6.81%, and 1.66%. Subsequent waterflooding increased final recovery to 57.04%, a total incremental of 14.93%. Thus, CO_2_–LFNA alternating flooding outperforms continuous CO_2_ flooding in homogeneous cores.

#### 3.3.2. Heterogeneous Physical Model Flooding Performance Evaluation

**Figure 11:**
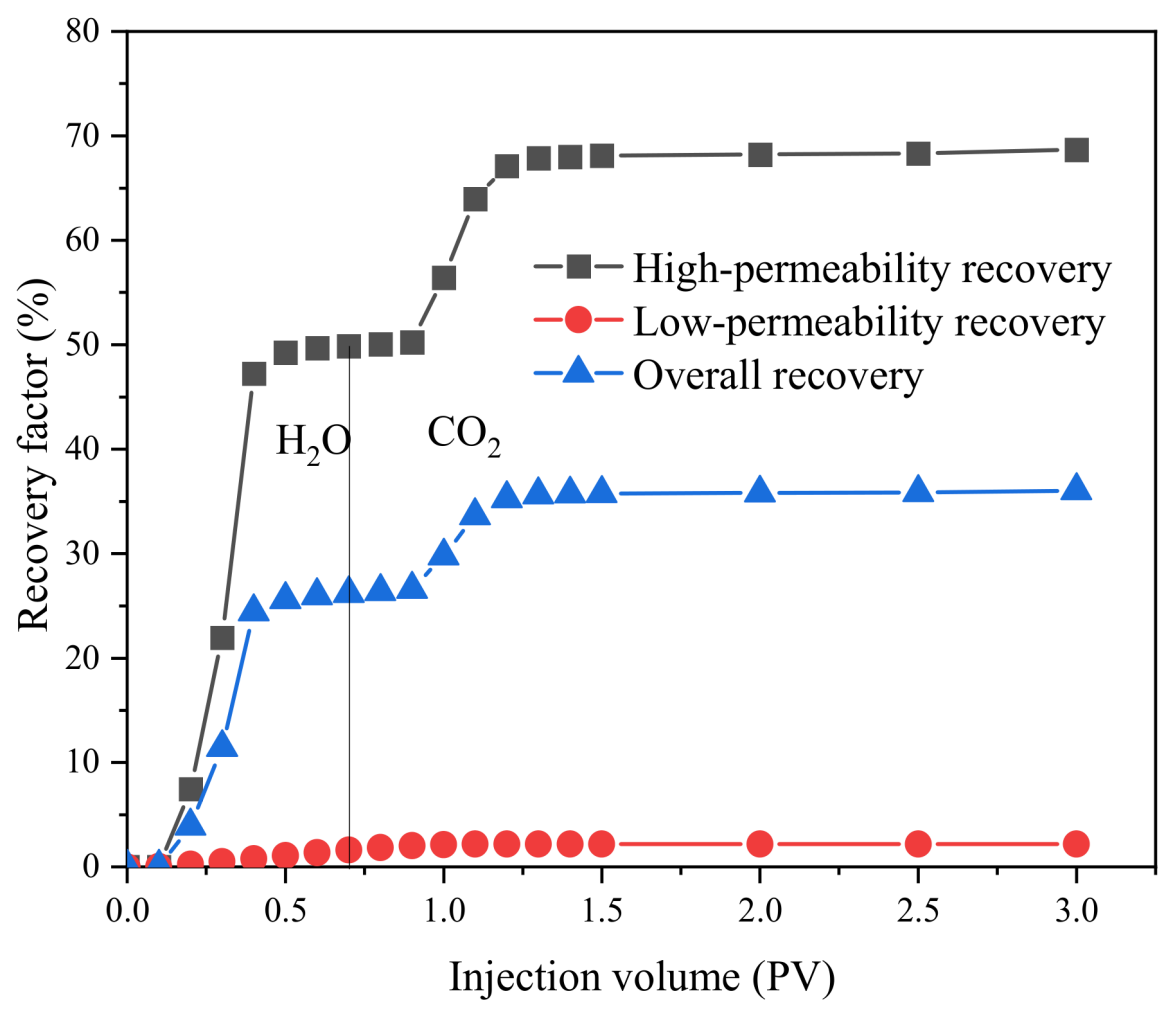
Continuous CO_2_ flooding after waterflooding with a permeability contrast of 5.

**Figure 12:**
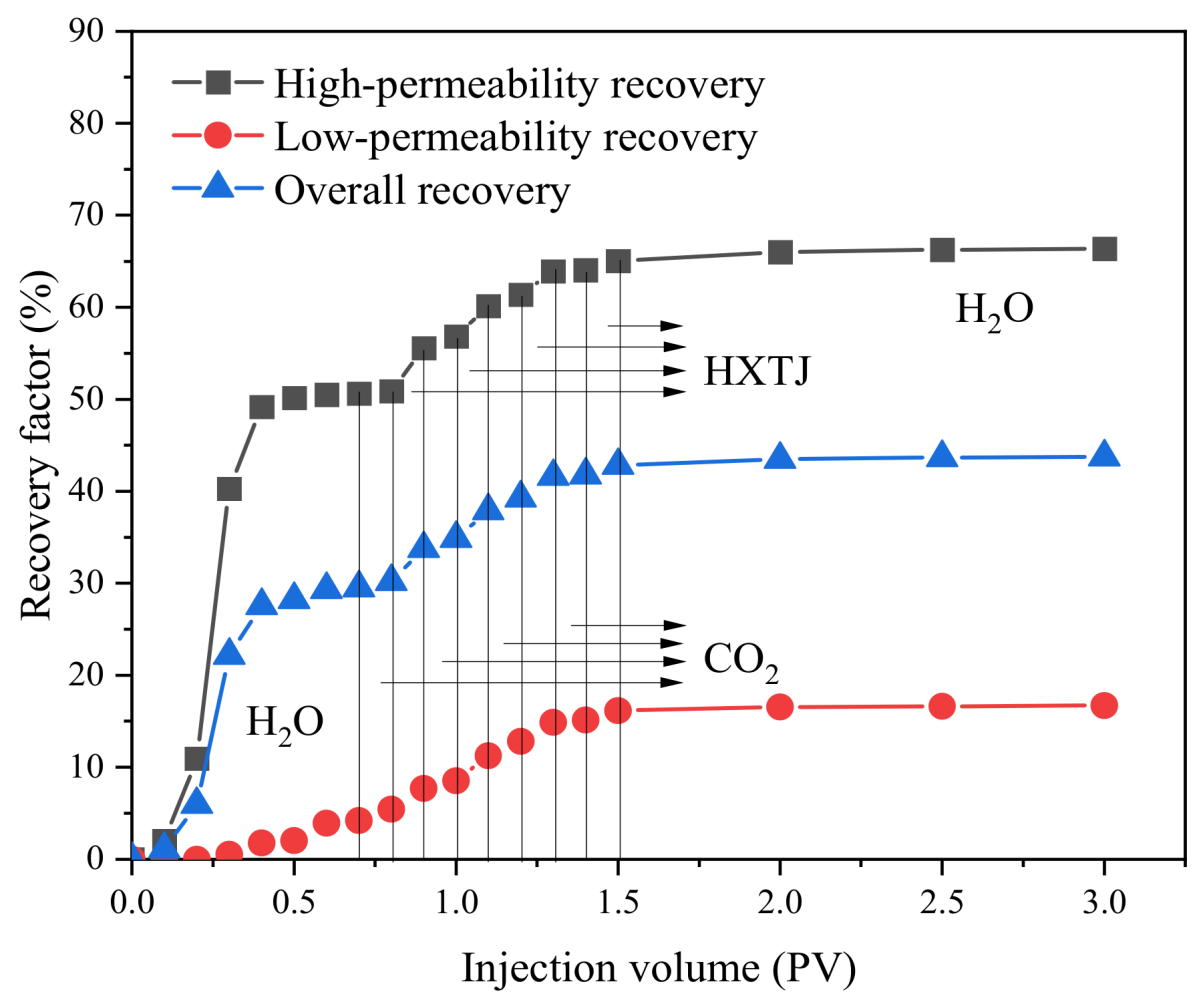
CO_2_–LFNA alternating flooding followed by waterflooding after waterflooding with a permeability contrast of 5.

At reservoir temperature (65 °C), pressure (13.6 MPa), and permeability contrast of 5, with constant injection rate of 0.1 mL/min, waterflooding to 98% water cut was followed by CO_2_ flooding or CO_2_–LFNA alternating flooding (Figure 11 and Figure 12).

As shown in Figure 10, in both waterflooding and continuous CO_2_ flooding, high-permeability core recovery consistently exceeded that of the low-permeability core. At 98% water cut, recoveries were 49.87% and 1.62% for high- and low-permeability cores, respectively, with a combined recovery of 26.20%. Subsequent CO_2_ flooding increased combined recovery by 9.85%, with only 0.57% incremental recovery from the low-permeability core, while the high-permeability core increased by 18.8%. This indicates that in heterogeneous tight reservoirs, post-waterflood CO_2_ flooding mainly enhances recovery from high-permeability channels, with significant gas channeling risk and limited ability to mobilize residual oil in low-permeability zones.

Figure 11 shows that at 98% water cut, high- and low-permeability recoveries were 50.62% and 4.21%, respectively, with a combined recovery of 29.50%. As CO_2_–LFNA alternating flooding proceeded, recoveries from both cores improved. For example, in Cycle 1, incremental recoveries were 4.93% and 3.47% for high- and low-permeability cores; Cycle 2 increments were 4.59% and 3.56%; Cycle 3 were 3.78% and 3.64%; and Cycle 4 were 1.13% and 1.27%. By the end of the fourth cycle, combined recovery increased by 13.30% over waterflooding. While additional cycles continued to improve recovery, the incremental benefit diminished notably in the fourth cycle. Subsequent waterflooding further increased high- and low-permeability recoveries by 1.31% and 0.54%, with combined recovery increasing by 0.93%. Overall, CO_2_–LFNA alternating flooding enhanced waterflood recovery by 15.74% and 12.48% for high- and low-permeability cores, respectively, with a combined incremental of 14.11%. This demonstrates that the LFNA low-IFT viscoelastic slug effectively plugs high-permeability layers, expands sweep volume, diverts flow to and mobilizes low-permeability zones, improves CO_2_ sweep efficiency, reduces gas channeling risk through high-permeability pathways, and enhances CO_2_ storage potential.

#### 3.3.3. Effect of Different Displacement Modes on Enhanced Recovery in Heterogeneous Cores

After waterflooding to 90% water cut, the effects of different displacement modes—continuous CO_2_ flooding, four-cycle 0.1 PV water–LFNA alternating flooding, and four-cycle 0.1 PV CO_2_–LFNA alternating flooding—on heterogeneous cores (permeability contrast 4.79–5.22) are summarized in Table 6.

With similar permeability contrasts (4.79–5.22), continuous CO_2_ flooding after waterflooding increased high-permeability recovery by 18.8% and combined recovery by 9.85%, but only marginally increased low-permeability recovery (0.57%). This indicates that in heterogeneous tight reservoirs, continuous CO_2_ flooding achieves moderate combined recovery improvement but has limited sweep, failing to effectively mobilize residual oil in lower-permeability zones, with significant gas channeling risk. In contrast, water–LFNA alternating flooding followed by waterflooding achieved better results, with a combined incremental recovery of 11.49%, notably higher than continuous CO_2_ flooding (9.85%). Importantly, low-permeability recovery increased significantly (8.64%), while high-permeability recovery also improved (13.69%). This demonstrates that LFNA preferentially enters high-permeability channels, effectively plugging them while enhancing oil-washing efficiency. These favorable effects are attributed to LFNA’s unique low IFT and viscoelasticity; LFNA also demonstrates effective water channeling control.

Further analysis of Table 7 reveals that post-waterflood CO_2_–LFNA alternating flooding followed by waterflooding yields the best performance, with a combined incremental recovery of 14.26%, surpassing water–LFNA alternating flooding. Both low- and high-permeability recoveries were significantly enhanced, reaching 12.49% and 15.74%, respectively. These results strongly demonstrate the synergistic effect between CO_2_ and LFNA, confirming that LFNA effectively plugs high-permeability channels, expands the sweep of CO_2_ and subsequent fluids, and possesses strong oil-washing capability. Using LFNA for CO_2_ channeling control is therefore feasible and highly effective.

## 4. Discussion

The core experimental observations of this work reveal the synergistic mechanism of LFNA during CO_2_–LFNA alternating flooding. The amphiphilic nanoparticle LQNA-3 forms electrostatic interactions and hydrogen-bond networks with the small-molecule viscosifier LFLM, which improves the viscoelasticity of the composite fluid without impairing its low-interfacial-tension property, as verified by rheology and interfacial-tension tests. Under reservoir-simulated salinity, temperature, and CO_2_-induced pH reduction conditions, LFNA retains its elastic-dominated viscoelastic behavior, which lays a foundation for its mobility-control performance in tight reservoirs. Core-flooding results confirm that the viscoelastic LFNA slug preferentially enters high-permeability channels and generates selective plugging rather than complete blockage. This flow diversion redirects subsequent CO_2_ into low-permeability zones to mobilize trapped residual oil. Meanwhile, its maintained low interfacial tension preserves oil-washing efficiency within high-permeability zones, so that both high- and low-permeability sections contribute to enhanced oil recovery. This accounts for why CO_2_–LFNA alternating flooding delivers higher incremental recovery than either continuous CO_2_ flooding or water–LFNA alternating flooding in heterogeneous core tests. For the amphiphilic nanoparticles used herein, the balance between hydrophilic moieties (sulfonic/carboxyl/hydroxyl groups) and hydrophobic alkyl chains governs cross-linking intensity within the LFNA transient network. Variations in carbon-chain length and hydrophilic group types alter non-covalent bonding strength between nanoparticles and LFLM molecules, further regulating the rheological properties of the final viscoelastic fluid.

The present study also reflects the practical characteristics and constraints of the LFNA system derived directly from the experimental data. According to the injectivity test results, no sharp pressure build-up occurred in tight cores (0.45–1.07 mD) during alternating injection, demonstrating that this small-molecule-based composite fluid avoids the poor injectivity issue common for high-molecular-weight polymer plugging agents. Nevertheless, the viscoelastic modulus of LFNA gradually declines with rising temperature and salinity within the tested range, even though tan δ remains below 1. The core-flooding experiments were performed with a fixed 0.1 PV slug size and limited alternating cycles; the incremental oil-recovery benefit clearly diminished after four alternating cycles, suggesting that excessive injection cycles cannot continuously improve production performance. All these experimental-based findings indicate that matching injection parameters with actual reservoir conditions is essential when applying LFNA for CO_2_ channeling mitigation, which should be considered in further laboratory and field-oriented investigation.

## 5. Methodology and Analysis

### 5.1. Materials and Instruments

Low-interfacial-tension small-molecule viscosifier (LFLM, molecular weight < 2000), amphiphilic nanomaterials (LQNA series, containing carboxyl, sulfonic, and hydroxyl hydrophilic groups, hydrophobic carbon-chain lengths of C8 and C12) were provided by Hubei Shengtang Petroleum Technology Co., Ltd. (Jingzhou, China). Natural cores (0.45–10.03 mD) and crude oil were obtained from the target reservoir. NaCl, CaCl_2_, KCl, MgCl_2_, NaHCO_3_, and Na_2_SO_4_ (analytical grade) were purchased from Tianjin Beilian Fine Chemicals Development Co., Ltd. (Tianjin, China). CO_2_ (purity 99.9%) was purchased from Anqiu Heng’an Gas Plant (Anqiu, China). MCR301 interfacial rheometer (Anton Paar, Graz, Austria), TX500C spinning drop interfacial tensiometer (Kino Industry Co., Ltd., Somerville, MA, USA), OCA50 fully automatic contact angle meter (Dataphysics, Filderstadt, Germany), high-temperature high-pressure core-flooding apparatus (National Key Laboratory of Low-Carbon Catalysis and CO_2_ Utilization), FA2004 electronic balance (Shanghai Sunny Hengping Scientific Instrument Co., Ltd., Shanghai, China), and HH-8 constant-temperature water bath (Changzhou Wantai Balance Instrument Co., Ltd., Changzhou, China) were used.

### 5.2. Experimental Methods

(1) Preparation of amphiphilic nanoparticle-containing low-interfacial-tension small-molecule viscosifying fluid.

Injection water (salinity 6788 mg/L, Na^+^ 1684, K^+^ 11, Mg^2+^ 75, Ca^2+^ 350, Cl^−^ 1033, HCO_3_^−^ 164, SO_4_^2−^ 3471 mg/L) was used. Required amounts of LFLM and LQNA were added and stirred at constant temperature to prepare the amphiphilic nanoparticle-containing low-interfacial-tension small-molecule viscosifying fluid (LFNA), then diluted to volume in volumetric flasks.

(2) Performance evaluation of amphiphilic nanoparticle-containing low-interfacial-tension small-molecule viscosifying fluid.

(a) Viscosity measurement

Viscosity of LFNA was measured using an MCR301 interfacial rheometer (Anton Paar GmbH, Graz, Austria). The test fluid was injected into a test cell placed in a constant-temperature bath, and the rotor height was adjusted until fully immersed in the fluid. The experimental temperature and shear rate (7 s^−1^) were set, and the stable viscosity value was recorded, with a viscosity error of 1 mPa·s ± 0.005 mPa·s.

(b) Viscoelasticity measurement

Viscoelasticity of LFNA was measured using an MCR301 rheometer. The viscoelastic behavior is expressed by phase angle δ (0° < δ < 90°), defined by the in-phase and out-of-phase components of the moduli: G′ = G_0_ cos δ, G″ = G_0_ sin δ (G′ storage modulus; G″ loss modulus). tan δ = G″/G′ characterizes viscoelasticity: tan δ ≥ 1 indicates predominantly viscous behavior; tan δ < 1 indicates predominantly elastic behavior [22]. Main procedures for viscoelasticity measurement are as follows: An amplitude-sweep test was first performed at 30 °C over a stress range of 0.0001 Pa^–1^ to determine the maximum stress within the linear viscoelastic region. A subsequent frequency-sweep test was carried out at 30 °C under a stress setting below this maximum value to select the target frequency. Finally, modulus measurement was conducted at 30 °C using the determined stress and frequency parameters. Storage modulus and loss modulus obtained from the modulus test were analyzed to identify whether the fluid exhibited elastic-dominant or viscous-dominant behavior.

(c) Oil/water interfacial tension measurement

Oil/water interfacial tension of LFNA was measured using a TX500C spinning drop interfacial tensiometer (Shanghai Zhongchen Digital Technical Equipment Co., Ltd., Shanghai, China). The rotation speed was set at 5000 r/min at the test temperature. Oil droplet morphology changes in the fluid were captured at different times, and droplet length and height were measured using software, which automatically calculated the interfacial tension at various times; the stable value was recorded.

(3) Reservoir environmental adaptability evaluation of LFNA

(a) Salt resistance evaluation

Using the target reservoir formation water salinity (80,000 mg/L, Na^+^ 9514, K^+^ 292, Mg^2+^ 57, Ca^2+^ 15,400, Cl^−^ 54,335, HCO_3_^−^ 100, SO_4_^2−^ 302 mg/L) as baseline, LFNA viscosity, interfacial tension, and viscoelasticity were measured at varying salinities (0–80,000 mg/L) to evaluate salinity adaptability.

(b) Temperature resistance evaluation

LFNA viscosity, interfacial tension, and viscoelasticity were measured over a temperature range of 25–85 °C to evaluate temperature resistance.

(c) pH tolerance evaluation

LFNA viscosity, interfacial tension, and viscoelasticity were measured over a pH range of 3.4–7.5 to evaluate pH tolerance.

(d) Injectivity evaluation of CO_2_–LFNA alternating synergistic flooding

Based on the target reservoir permeability range, different permeability cores were selected to evaluate the injectivity of CO_2_–LFNA alternating flooding at reservoir temperature (65 °C).

① Saturation with formation water: Selected dry cores were weighed, vacuumed, and pressure-saturated with formation water. After saturation, the wet weight was recorded. Pore volume (PV) was calculated from the weight difference and formation water density.

② Waterflooding: Saturated cores were placed in a core holder with confining pressure applied. Injection water was used to flush inlet lines, and waterflooding was conducted at 0.1 mL/min until outlet flow rate and inlet pressure stabilized; pressure and related data were recorded to calculate water-phase permeability.

③ CO_2_–LFNA alternating flooding: After waterflooding pressure stabilized, CO_2_/LFNA alternating flooding (slug size tentatively 0.1 PV each) was performed at 0.1 mL/min (at least three alternation cycles). Pressure changes during injection were recorded until stabilization. Injectivity was evaluated based on injection pressure variations [14].

(4) Physical model flooding performance evaluation with different displacement fluids

(a) Homogeneous physical model flooding performance evaluation

At reservoir temperature (65 °C) and constant reservoir pressure (13 MPa), CO_2_ flooding, water–LFNA alternating flooding, and CO_2_–LFNA alternating flooding were conducted on cores with similar permeability to evaluate displacement efficiency.

① Waterflooding: Cores were saturated with formation water and oil to establish irreducible water saturation. Waterflooding was conducted at 0.1 mL/min under reservoir temperature and pressure until 98% water cut; injection parameters were recorded, and waterflood recovery was calculated.

② CO_2_ flooding: CO_2_ was injected at 0.1 mL/min until no oil was produced from the outlet; incremental recovery was calculated.

③ Water–LFNA alternating flooding: Repeat step ①; then water–LFNA alternating flooding (0.1 PV slug, 3–4 alternations) was performed at 0.1 mL/min until no oil was produced. Incremental and subsequent waterflood recoveries were calculated.

④ CO_2_–LFNA alternating flooding: Repeat step ①; then CO_2_/LFNA alternating flooding (0.1 PV slug, 3–4 alternations) was performed at 0.1 mL/min, followed by subsequent waterflooding. Injection parameters were recorded to obtain recovery vs. cumulative injection curves and incremental recovery data.

(b) Heterogeneous physical model flooding performance evaluation

At reservoir temperature and constant reservoir pressure, reservoir heterogeneity was simulated using parallel high- and low-permeability cores. CO_2_ flooding, water–LFNA flooding, and CO_2_–LFNA alternating flooding were evaluated for displacement efficiency. Experimental procedures followed those described for homogeneous models [19].

## 6. Conclusions

(1) The viscous fluid formulated with low-interfacial-tension small-molecule viscosifier LFLM and amphiphilic nanoparticles LQNA exhibits good viscosifying ability (5.82 mPa·s), low interfacial tension (0.0296 mN/m), and viscoelasticity (tan δ = 0.791 < 1, predominantly elastic), with a simple formulation (0.4 wt% LFLM + 0.01 wt% LQNA-3). It is designated as an amphiphilic nanoparticle-containing low-interfacial-tension viscoelastic fluid (LFNA).

(2) LFNA demonstrates good temperature and salt resistance, as well as adaptability to pH reduction caused by CO_2_ dissolution in saline water. It exhibits good injectivity in tight reservoir cores (Kg = 0.45–1.07 mD) and selective permeability for plugging.

(3) Following waterflooding, CO_2_–LFNA alternating flooding outperforms post-waterflood continuous CO_2_ flooding in enhancing oil recovery in both homogeneous and heterogeneous models. In the CO_2_–LFNA WAG process, the LFNA slug effectively plugs high-permeability channels and expands sweep volume, efficiently mobilizing residual oil in low-permeability channels with a recovery factor of 12.49%, while its low interfacial tension maintains good oil-washing efficiency in high-permeability channels, achieving a recovery factor of 15.74%.

(4) In heterogeneous models, CO_2_–LFNA alternating flooding exhibits a clear synergistic oil recovery effect, with a combined EOR after waterflooding of 14.26%, surpassing water–LFNA alternating flooding (11.49%) and continuous CO_2_ flooding (9.85%). These results indicate that LFNA effectively plugs high-permeability channels, improves the sweep efficiency of CO_2_ and subsequent fluids, and possesses strong oil-washing capability. The use of LFNA for CO_2_ flooding channeling control is feasible, significantly effective, and beneficial for CO_2_ storage and carbon reduction.

## Figures and Tables

**Table 1:** Effect of amphiphilic nanoparticles on viscosity and oil/water interfacial tension of low-interfacial-tension small-molecule viscosifying fluid (47 °C).

Fluid Composition (Mass %)	LFLM (0.4%)	LFLM + 0.01% LQNA-1 (C8, Carboxyl)	LFLM + 0.01% LQNA-2 (C12, Carboxyl)	LFLM + 0.01% LQNA-3 (C8, Sulfonic)	LFLM + 0.01% LQNA-4 (C12, Sulfonic)
Viscosity (mPa·s)	4.12	5.67	5.44	5.82	5.49
IFT (10^−2^ mN/m)	2.83	3.11	3.18	2.96	3.07

**Table 2:** Effect of LQNA-3 concentration on viscosity, oil/water IFT, and viscoelasticity of LFNA (47 °C).

Concentration (%)	Viscosity (mPa·s)	IFT (mN/m)	tan δ
0	4.12	0.02833	0.878
0.01	5.82	0.02961	0.791
0.02	5.42	0.02956	0.802
0.03	5.01	0.03025	0.834

**Table 3:** Effect of salinity on the viscoelasticity of LFNA.

Salinity/mg·L^−1^	tan δ	Behavior
0	0.812	Elastic-dominant
6788	0.791	Elastic-dominant
16,570	0.853	Elastic-dominant
34,140	0.874	Elastic-dominant
50,710	0.917	Elastic-dominant
80,000	0.952	Elastic-dominant

**Table 4:** Effect of pH on the viscoelasticity of LFNA.

pH Value	tan δ	Behavior
7.5	0.791	Elastic-dominant
6.1	0.821	Elastic-dominant
5.4	0.875	Elastic-dominant
3.4	0.904	Elastic-dominant

**Table 5:** Effect of temperature on the viscoelasticity of LFNA.

Temperature/°C	tan δ	Behavior
25	0.50	Elastic-dominant
47	0.79	Elastic-dominant
65	0.90	Elastic-dominant
85	0.93	Elastic-dominant

**Table 6:** Comparison of different post-waterflood displacement modes in heterogeneous physical models.

Displacement Mode	Kg/ (mD)	Soi (%)	Recovery at 98% Water Cut (%)			EOR%		
			Low-Perm	High-Perm	Combined	Low-Perm	High-Perm	Combined
CO_2_ flooding	1.11	50.01	1.62	49.87	26.20	0.57	18.8	9.85
	5.52	51.25						
Water–LFNA WAG + subsequent waterflood	1.31	56.17	7.50	48.93	30.93	8.64	13.69	11.49
	6.27	61.03						
CO_2_–LFNA WAG + subsequent waterflood	1.13	51.20	4.21	50.62	29.50	12.49	15.74	14.26
	5.90	52.3						

**Table 7:** Summary of key experimental input parameters for core-flooding and fluid-performance tests.

Parameter Category	Parameter Details	Value/Setting
Fluid formula	Base injection water salinity	6788 mg/L
	Salinity test range	0–80,000 mg/L
	pH test range	3.4–7.5
Rheological test	Steady-state viscosity shear rate	7 s^−1^
Interfacial tension test	Rotation speed of spinning drop tensiometer	5000 r/min
Core-flooding general conditions	Injection rate	0.1 mL/min
	Reservoir simulation temperature	65 °C
	Reservoir simulation pressure	13 MPa
	Slug volume for alternating injection	0.1 PV
	Number of alternating cycles	3–4 cycles
Core property	Permeability of tight cores	0.45–10.03 mD

## Data Availability

The original contributions presented in this study are included in the article. Further inquiries can be directed to the corresponding authors.
